# The Clinical Significance of Glycosuria in Pregnant Women

**Published:** 1910-03-05

**Authors:** 


### THE CLINICAL SIGNIFICANCE OF GLYCOSURIA IN PREGNANT WOMEN."

A positive reaction with Fehling's solution during pregnancy does not necessarily indicate the existence of diabetes, but is usually due to lactosuria, or to transient, alimentary, or recurrent glycosuria. In such cases it is imperative to determine whether the sugar occurs as lactose or glucose, for lactosuria is without clinical significance and is probably associated with premature activity of the breasts. The fermentation and the phenylhydrazine tests should therefore be applied.

The significance of glycosuria is not so clear. If alimentary in character, it may be disregarded with impunity. Otherwise, it may be of the transient or recurrent variety, or may indicate the existence of true diabetes. If glycosuria appears late in pregnancy, does not exceed 2 per cent. in amount, and is not accompanied by symptoms, it is probably transient and may disappear spontaneously at any time, or persist until the end of pregnancy. In either event it is usually of slight clinical significance, and merely indicates that the patient should be carefully watched. If the sugar appears in pregnancy and in large amounts, the condition is more serious.

Pregnancy may occur in diabetic women, or diabetes may become manifest during pregnancy. Either is a serious complication, although the prognosis is not so alarming as is frequently stated; many patients do perfectly well, while a smaller proportion die in coma, or collapse at the end of pregnancy, or during or shortly after labour. If the output of sugar is large and cannot be controlled, or at least markedly diminished, by suitable dietetic and medicinal treatment, the induction of abortion or premature labour is indicated even in the absence of serious symptoms, and much more so when they are present. In pregnant women, as in any other cases of diabetes, it is important to remember that the actual presence of sugar in the urine is not a sign of bad prognosis unless there is at the same time evidence of acidosis. This is indicated by the presence in the urine of di-acetic acid and acetone, in addition to sugar. It is much more important to apply the sodium nitroprusside test to the urine in cases of this kind, therefore, than it is to rely on Fehling's test alone.

